# Observations upon the Importance and Value of a Knowledge of the Collateral Branches of Medicine and Surgery in Connection with Dentistry

**Published:** 1848-01

**Authors:** James Robinson

**Affiliations:** London.


THE AMERICAN

### Journal of Dental Science.

Vol. VIII.] JANUARY, 1848. [No. 2.

### ARTICLE I.

*Observations upon the Importance and Value of a Knowledge of the Collateral Branches of Medicine and Surgery in connection with Dentistry.* By James Robinson, D. D. S., London.

[Continued from p. 33.]

Having in former papers disposed of materia medica, therapeutics and botany, we are naturally led to another branch of knowledge essential to a medico-dental education, and without which, our acquaintance with these would be comparatively useless—we allude to pharmacy or the art of preparing, preserving, combining and compounding the different substances employed in the practice of physic.

A knowledge of this subject is of greater importance as the substances of which it treats not only vary considerably in their characters, effects and modes of combination, but are derived alike from the animal, vegetable and mineral kingdoms. This knowledge, however, must not be supposed to consist merely in an acquaintance with the pharmacopiæ, (however perfect that acquaintance may be,) which simply teaches the mode of preparing from fixed formula, certain medicines, which the apothecary and chemist are supposed to keep ready for use, but in a perfect acquaintance with the nature of the different medicines and all the various modes in which they may advantageously be employed, either alone or in combination with each other, for as it is rarely the case that a medicine finds its way either into the materia medica or pharmacopiæ, till its value has been stamped by its long and successful exhibition, a medical man, whether a general practitioner or dentist, must be content to follow in the beaten track, and dwell in (medical) decencies forever, unless he be prepared to think, reason and act for himself, and this he can only do by a perfect knowledge of the means at his command, and of the best and most judicious way of employing them. He may be perfectly acquainted with the mysteries of ℞ of rhubarb, have mastered the pons assinorum of Griffith's mixture, may be able to talk learnedly of decomposition, double decomposition and precipitation, and yet be as ignorant of the principles of pharmacy as an elephant of the polka. Young gentlemen *go through* the pharmacopiæ with a *grinder*, are parrotted in the different compositions and formulæ, learn to run over the chemical changes as glibly, as an Eaton boy his hic-hac-hoc, and yet after all, know nothing whatever of the subject.

To really understand pharmacy, a knowledge of botany, zoology and mineralogy is essential, while to determine the qualities and modes of action of the different pharmaceuticals, and understand the laws by which their combinations with, and actions on each other are governed, we must call in the aid of chemistry.

The mineral kingdom contributes largely to medicine—simples, as they are called, are in a great measure banished from practice as being less certain and determinate in their action and more likely to suffer deterioration from time, and a variety of extraneous circumstances, and chemical and concentrated remedies are the fashion of the day. Thus we have the various preparations of iron, quicksilver, antimony and iodine, &c. The preparations of morphia, narcotine, veratrine and strychnine. We have the principle of the black-cherry, water of the bitter almond concentrated in hydroscyanic acid, and the active properties of the nauseous cinchona into the elegant and more definite quinine for all these—for the chlorates of lime and potass, the hydrodate of potass, and other active and valuable remedial agents, we are indebted to chemical research, and scientific experimental practice.

It must, however, be kept in mind, that while chemical and concentrated medicines are, if scientifically employed, more definite and certain in their operation than those of a more simple nature, they are at the same time more dangerous to handle and more liable to have their actions interfered and contravened by ignorant and unscientific management.

Thus, then, while pharmacy technically speaking may be considered to mean the art of compounding medicines, it has really a much higher mission, forming at least *one* of the most important branches of medical education, and so dependent on, and mixed up with the others, that whatever department of medicine a practitioner may devote himself to, an intimate acquaintance with its principles and practice is essential to his success, for although a knowledge of botany, materia medica and therapeutics, forms the base on which he will have to act, such knowledge would be comparatively useless, unless he were acquainted with the different modes in which the various remedial agents may be combined with, and act upon each other. He may practice, it is true, but his practice will be unscientific, unsatisfactory and unsafe.

We will suppose, that in a case (which, though taken from general practice, will exemplify our position) requiring a certain description and amount of tonic, a practitioner ignorant of pharmaceutical chemistry prescribes the protoxyde of iron, in conjunction with a carbonate of soda or potass, what would be the result? With the first alkali, decomposition would take place, and produce a tonic, certainly, (the carbonate of iron,) but one of so inert and questionable a nature as to lie under the suspicion of being useless, while with the latter alkali potass he would have a ferrocuret of potassium, a deleterious if not fatal poison. We again suppose, (and we have seen such a thing,) a prescription intended to act as a purgative, contained tartaric acid and potass in conjunction with the sulphate of iron—the result would be a tonic which would, at least, frustrate the intentions of the medical attendant, and *might*, under certain conditions, produce even fatal effects.

It is clear, then, that while a knowledge of pharmacy is essential to the medical practitioner of every grade and of every kind, chemistry is one of its most important adjuncts and that without it, our knowledge must be imperfect, and our practice empyrical, subject to frequent ridiculous and in some cases fatal mistakes.

It may be asserted, that the dentist has no business with the general treatment of his patient, but conceding this, does it follow that he can practice scientifically *his* branch of the profession if his knowledge be so disjointed that it begins and ends with the mouth; and *may* not cases arise in which from the inability of a patient to remunerate *two* separate practitioners, he may be *obliged* to take the case into his own hands? or, again, if ignorant of medical science generally, how can he be certain that the applications he prescribes for the mouth, may not have a dangerous or even fatal effect upon the body? Such things *have* occurred, and as the dental practitioners are better educated than they are wont to be, will occur again and again.

Chemistry, however, has other and important connections with medical science, it teaches us the nature of different bodies, enabling us to separate the various elements of which they are composed into elemental particles, to ascertain the different proportions of their component parts, and the action not only on each other, but on others with which they may be brought into contact.

Thus, while this science shows us the nature and proportions (at least as far as our present knowledge extends) of all bodies, natural philosophy, or physics, examines and ascertains the reciprocal action of matter in masses, so that it is as impossible to separate chemistry from physics as it would be to understand pharmacy without its aid; neither the one or the other can be properly understood, unless chemistry forms one of its fundamental studies.

But chemistry is divided into seven or eight branches, which, though it is not our intention to investigate in this paper, we will enumerate. Thus, we have meteorological, geological and agricultural chemistry, then comes medical chemistry, which again is subdivided into physiological chemistry, or that which considers the changes produced in animal matter in the operations of healthy life—pathological chemistry which treats of the changes caused in animal substances by disease, or in other words, diseased changes in the structure of parts. Therapeution, pharmaceutical chemistry which teaches the nature of different medical remedies, the means of preparing and preserving them intact. And hygietic chemistry, by which we learn the means of arranging our habitations in the measure most advantageous to health, and are taught to select food the best suited to our wants and constitutions.

In viewing this brief outline of the different branches of chemical science, its important connection with all the arts and sciences is self-evident, and why dental surgery which should embrace not only the whole range of medical education, but in addition requires an intimate acquaintance with the laws of mechanism, should be exempt from its influence is a question beyond our powers to answer.

To apply chemical knowledge with advantage to the dental art, the practitioner must be in possession of its theories and the laws by which they are regulated, but more especially conversant with those branches of the science on which he will have to call most frequently for assistance, viz. physiological, pathological, therapeutic and mineralogical chemistry.

A knowledge of the nature of the enamel, of the saliva, of the tartar, and various concretions occasionally found in the mouth, have originated in chemical research—and the professor of the dental art who is ignorant on these subjects will cut a sorry figure, and search in vain for the explanation of phenomena that would be perfectly clear to the scientific practitioner, while his remedial means will be curtailed and uncertain.

We will take an example, and suppose that a portion of the inferior maxillary bone has become affected through disease of the dental organs, situated in its immediate vicinity, here we should have occasion for the exercise of our knowledge of pathological chemistry.

Again, in the introduction of metals into the mouth, either for the purpose of stopping teeth, or replacing them with natural or artificial ones, an acquaintance with the laws of chemistry is essentially necessary to enable us to guard against any deleterious action on the parts, or on the system generally, that might arise from their decomposition, and the formation of some new compound, pernicious to health. This may be exemplified by using an improper quantity of copper as an alloy for the bases of artificial teeth, or by the employment of silver rivets to gold plates, in either case the acrid secretions of the mouth produce a constant series of galvanic actions, and the patient is never free from a nauseous and disagreeable taste of copper, more particularly when the stomach is empty, but more particularly in the composition of the various pastes or cements used to plaster up the teeth, and which from their being composed of a combination, or in other words, an amalgam of silver and quicksilver, act deleteriously not only on the teeth, but have, in more than one instance, caused salivation. Can any one who is not possessed of chemical and pathological knowledge, venture to employ arsenic in his treatment of the teeth? and yet there are cases in which even this virulent poison may, in the hands of a judicious practitioner, be valuable.

Unquestionably, then, chemistry forms an important branch of dental education; without its aid, the practitioner will be constantly making the most unscientific and ridiculous mistakes, inimical alike to the comfort and safety of his patient and his own reputation, his resources will be cramped and confined, and instead of having the power of thinking and reasoning for himself, and adapting his remedial means to the emergencies of each particular case with scientific skill and judgment, he will be bound down to precedents, fixed to a certain routine of practice, and, however successful in a pecuniary view, unable to attain scientific eminence, or to hold a place among the benefactors of his profession and mankind; he may be a tooth-drawer, may be able to stop, scale the teeth, or even remedy their morbid arrangements, but he will never be a *scientific* professor of dental surgery.

The science of comparative anatomy although not one that can be strictly considered a legitimate branch of dental education, is, as being of immense importance to the study of physiology, of great assistance to the dentist, and Haller justly observes, "that physiology has been more illustrated by comparative anatomy, than by the dissection of the human body." It is to this science that we are indebted for the classification of animals according to their anatomical structure; it is by its aid that we are enabled to ascertain the habits and the varieties in the construction of different animals which in this way afford data on which by inductive reasoning much benefit has been conferred on the human race; in short, it forms one of the links of the chain of natural philosophy which the more liberal opinion of modern times has, at least, tacitly considered as essential to the scientific practitioner of every branch of the dental art.

This applies generally to comparative anatomy, but when we consider the specific results that have occurred to the science of odontologia, the philosophical value attached to the teeth, as the most important means of classification in natural history, and that much has been done by microscopic investigation of their structure, to raise the art of dentistry, we must allow that comparative anatomy is of great importance in the education of the dentist.

It is generally acknowledged that professional men should, independently of their particular line of study, have a liberal and finished education, and what would be thought of him who was unacquainted with a science connected, however remotely, with his profession. Education is now so common that a gentleman in even respectable practice will meet with many patients having considerable knowledge on these subjects, and who would have little confidence in a professional attendant possessed of less knowledge than themselves on any subject connected with natural philosophy.

